# The development of national injury prevention policy in the Australian health sector: and the unmet challenges of participation and implementation

**DOI:** 10.1186/1743-8462-3-11

**Published:** 2006-10-23

**Authors:** Rebecca Mitchell, Rod McClure

**Affiliations:** 1 NSW Injury Risk Management Research Centre, University of New South Wales, Sydney, Australia; 2 School of Medicine, Griffith University, Queensland, Australia

## Abstract

For the last 20 years injury prevention policy in Australia has been hampered by poor consultation practices, limited stakeholder involvement, inadequate allocation of resources, poor implementation, and an absence of performance measures. This paper describes the development of injury prevention policy in Australia from its beginnings in 1981 to the current day and considers what measures should be undertaken to create an effective platform for the reduction of the burden of injury in Australia.

The *National Injury Prevention and Safety Promotion Plan 2004–2014*, released in 2005, needs to be supported by a whole of government commitment to the reduction of injury. The Council of Australian Governments would be an ideal forum to monitor progress, supported by a cross-government Ministerial Council.

## Background

Approximately 7,800 Australians die each year from injury [1]. Evidence suggests that there are $1.3 trillion of potential health gains to be made from reducing injuries alone [2]. Reducing injuries in Australia by just under one-third would equal savings of over $370 billion which is greater than Australia's total net foreign debt [2].

In July 2005, the Australian Health Ministers approved release of the *National Injury Prevention and Safety Promotion Plan 2004–2014*. The policy is the culmination of activity within the Australian health sector that spans two decades. In this paper, we describe the critical features of the period of national injury prevention policy development since the Better Health Commission report of 1986, and argue that low levels of community participation and inadequate government commitment to implementation has compromised the effectiveness of this policy. We conclude that unless there is a genuine cross sectoral involvement in an adequately resourced whole of government commitment to implementing the *National Injury Prevention and Safety Promotion Plan 2004–2014 *then this health-based national injury prevention policy will have no impact on the population level indicators of injury in Australia.

## The history of national injury prevention policy development within the health sector in Australia

The development of national injury prevention policy in Australia (Table 1) began in 1981 when the World Health Organization (WHO) published the *Global Strategy for Health for All by the Year 2000 *[3]. In response to this call for all WHO Member States to develop national policies, strategies and action plans to improve health and to monitor the effectiveness of their progress against specified actions, the then Federal Minister for Health, Dr Neal Blewett, created the Better Health Commission in 1985 [4]. The Commission was asked to report on the current health status of the Australian population and to focus on the development of health-related policy relating to the prevention of disease and injury [5,6].

**Table 1:** Development of injury prevention policy in Australia by the health sector

**Year**	**Initiative**
1981	*Global Strategy for Health for All by the Year 2000 *published by World Health Organization.
1985	Better Health Commission established to respond to WHO initiative.
1986	*Looking Forward to Better Health *published by the Better Health Commission.
1987	Health Targets and Implementation (Health for All) Committee established.
1988	*Health for all Australians. Report of the Health Targets and Implementation (Health for All) Committee to Australian Health Ministers *published.
1988	Establishment of the National Better Health Program.
1991	Review of the National Better Health Program.
1992	Medicare Agreements Act 1992 requires Commonwealth and State and Territory Governments to have developed national health goals and targets by 30 June 1994.
1993	*Goals and Targets for Australia's Health in the Year 2000 and Beyond *published.
1993	National Health Summit held.
1994	*Better Health Outcomes for Australia *published and Better Health Outcomes Overseeing Committee established.
1996	National Health Priority Area initiative established.
1996	National Public Health Partnership Group formed.
1997	National Injury Prevention Advisory Council established.
1997	*National Health Priority Area: Injury Prevention and Control *report published.
1999	*Directions in Injury Prevention. Report 1: Research Needs *and *Directions in Injury Prevention. Report 2: Injury Prevention Interventions Good Buys for the Next Decade *published.
2000	Strategic Injury Prevention Partnership established as a subcommittee of NPHPG.
2001	*National Injury Prevention Plan Priorities for 2001–2003 *and *National Injury Prevention Plan Priorities for 2001–2003 Implementation Plan *published.
2004	Evaluation of the National Injury Prevention Plan Priorities for 2001–2003.
2005	*National Injury Prevention and Safety Promotion Plan 2004–2014 *endorsed.
2005	*National Aboriginal and Torres Strait Islander Safety Promotion Plan 2004–2014 *endorsed.
2005	*National Falls Prevention for Older People Plan: 2004 onwards *endorsed.

In 1986, the Better Health Commission published *Looking Forward to Better Health *in three volumes [5,7,8]. Among the Commission's recommendations were that major prevention activities should concentrate on the three areas of cardiovascular disease, nutrition, and injury. Following the release of the Better Health Commission report, the Australian Health Ministers Advisory Council (AHMAC) established the Health Target and Implementation Committee (HTIC) in 1987 to provide advice regarding how the recommendations from the Better Health Commission report could be implemented [9]. In 1988, the HTIC published *Health for All Australians *[10] which outlined goals and targets in three main areas: (1) population groups; (2) major causes of illness and death (including injury); and (3) risk factors. The report also included five priority health areas for preventive action of which injury was one. The National Better Health Program (NBHP) was established in 1988 to oversee the implementation of the strategies outlined in the HTIC report.

Four years after its creation, a review of the NBHP was conducted and found that while progress had been made in some areas, there were limitations of the approach to the goals and targets suggested in the *Health for all Australians *report [6,11]. The review identified that the goals and targets listed in the report had not been widely adopted. It highlighted the strong need for the health system to be fully engaged in both the identification and monitoring of any health targets, in the development of accountability measures, and in the development of strategies for addressing both the social and environmental determinants of health [4,12,13]. As an added stimulus for the development of a consistent national approach to health goals and targets, in 1992 the Medicare Agreements Act required the Commonwealth and the State and Territory Governments to have developed national health goals and targets by 30 June 1994 [14].

*Goals and targets for Australia's Health in the year 2000 and beyond *was commissioned by the Federal Government from academics in the Department of Public Health at the University of Sydney and it was published in 1993 [13]. This report revised the goals and targets of *Health for all Australians *into four principal areas: (1) mortality, morbidity and quality of life; (2) healthy lifestyles and risk factors; (3) health literacy and life skills; and (4) healthy environments.

*Goals and targets for Australia's Health in the year 2000 and beyond *adopted a more social view of health than previous health-related goal and target setting reports. It contained over 100 goals and 600 specific targets, 76 targets related to injury prevention, including transport-related injuries, suicide, interpersonal violence, injuries in a residential setting, and product- and sport-related injuries. Targets for safe behaviours, such as wearing helmets while bicycling and mouthguard wearing during contact sports, were also set [6,13]. Reviewing the report at a National Health Summit in April 1993, the Australian Health Ministers agreed that the goals and targets outlined in the report should be included within a broader framework of national health policy [4,11,12]. Subsequently, a joint working group of AHMAC and the National Health and Medical Research Council (NHMRC) was established to identify some initial national health focus areas for action. The working group identified four priority health areas: (1) cardiovascular disease; (2) cancer; (3) injury; and (4) mental health, and implementation groups were established for these areas.

In 1994, the *Better Health Outcomes for Australians *report was published which brought together the work of the four implementation groups[14]. This report provided a number of goals, strategies, and indicators for these four priority health areas, of which 22 goals and 29 targets related directly to injury prevention, in the areas of transport-related injuries, work-related injury, falls in older persons and in young children, product- and sport-related injury, interpersonal violence, poisoning, burns and scalds, and drowning. This report also included two goals related to post injury management. Following the release of the report, the AHMAC established the Better Health Outcomes Overseeing Committee (BHOOC) [4,11]. One year later, the BHOOC reviewed the national health goals and targets process and identified a number of issues. It appeared that the complexity of the goals and targets were a problem, the number of indicators were too large (over 140 indicators across four health areas), and no national reporting requirements existed [4]. Following this review, the National Health Priority Area (NHPA) initiative was established by the Australian Health Ministers in July 1996 [4].

In 1996, AHMAC agreed that a report should be prepared on each of the four health priority areas every two years to provide an overview of the burden and the key issues relating to each area [15]. The AHMAC also agreed that diabetes should be added to the health priority areas, making a total of five priority areas and that a National Public Health Partnership Group (NPHPG) should be formed in an attempt to develop a national coordinated approach to public health. In 1997, the first report on Injury Prevention and Control was published as part of the series on national health priority areas [15]. In the same year, the National Injury Prevention Advisory Council (NIPAC) was established to provide independent advice to the then Commonwealth Department of Health and Aged Care (DHAC) and to Health Ministers on appropriate strategies to reduce the incidence and severity of injury in Australia. Members of NIPAC included representatives from DHAC, the state and territory health departments, injury prevention researchers, and practitioners.

In 1999, NIPAC and the DHAC produced two reports describing proposed directions in injury prevention, including research needs and effective interventions for injury prevention [16,17]. These reports provided an overview of injury in Australia, described known effective injury prevention measures, and outlined opportunities for investment in injury prevention strategies that were likely to be the most cost-effective. During this period, NIPAC developed the first National Injury Prevention Plan, which contained four priority areas for injury prevention: (1) falls in older people; (2) falls in children aged 14 years or less; (3) drowning and near-drowning; and (4) poisoning in children aged four years or less.

The national plan and its accompanying implementation plan were not published until 2001 [18,19] following the demise of the NIPAC and the formation of the Strategic Injury Prevention Partnership (SIPP) in August 2000. SIPP was developed as a subcommittee of the NPHPG, and consisted of representatives of DHAC, State and Territory Health Departments, the Australian Institute of Health and Welfare, the Consumer Safety Unit of the Australian Treasury Department, and the Australian Injury Prevention Network.

In 1999, a review of the NHPA initiative found wide support for the continuation of the initiative and a number of recommendations were made, including the need to give greater emphasis to the identification and promotion of evidence-based prevention strategies, and the need for the provision of timely and appropriate indicators to monitor performance and implementation of NHPA strategies [4]. Following this review, asthma, in August 1999 and arthritis and musculoskeletal conditions in July 2002 became the sixth and seventh national health priority areas, respectively [4]. In 2004, a review of the first national injury prevention plan was released. Although the terms of reference of this review were limited, the finding of the review concluded that the plan was created following ineffective consultation, was confined only to the areas of injury prevention over which the health sector had influence, did not fully engage injury prevention stakeholders, lacked resources for implementation, appropriate performance measures to gauge progress were absent, and thus the plan was unlikely to have any significant effect on rates of injury-related morbidity and mortality in Australia [20].

In 2005, SIPP submitted a ten year National Plan for Injury Prevention and Safety Promotion to the Australian health ministers. The *National Injury Prevention and Safety Promotion Plan 2004–2014 *[21] was largely based on both the New Zealand and Canadian Injury Prevention Strategies and identifies two main goals – achieving a positive safety culture and creating safe environments, ten underlying principles, eight priority areas and 71 priority activities, rather than setting specific targets. It represents a return to a population-based focus and includes as its priorities: children, youth and young adults, adults, older people, rural and remote populations, and Aboriginal and Torres Strait Islander peoples. One risk factor, alcohol, is identified as a priority area, along with the core feature of the establishment and maintenance of a national strategic framework for the prevention of injuries. The plan was endorsed by the Health Ministers and SIPP was immediately dissolved.

## The Future of the National Injury Prevention and Safety Promotion Plan 2004–2014

While not limited to government activity, the term policy tends to be defined as 'government action'[22] and more specifically, 'an action which employs governmental authority to commit resources in support of preferred values' [23]. Although committees have been formed, policy documents written and reviews undertaken, as demonstrated in the above section, both the 'action' and 'commit resources' components of the definition have largely been absent from the 20 year history of injury prevention policy in Australia.

While injuries are treated within the health system, the risk factors for injury (eg. environmental, social or object-specific) and the creation of legislation and standards that aim to prevent injuries largely lie outside the jurisdiction of the health sector. For instance, in Australia many policy and legislative factors can affect drowning incidence rates and these are beyond the control of any one government or non-government agency. For example, in NSW, the NSW Swimming Pool Act 1992 and its associated Regulation 1998 are enforced by the NSW Department of Local Government; safe boating is regulated by NSW Maritime and the NSW Water Police; the promotion of water and surf safety is conducted by the NSW Department of Sport and Recreation, Surf Life Saving, Royal Life Saving, Austswim, and the Australian and Professional Ocean Lifeguards Association (APOLA); rural water safety and dam safety is promoted by Farmsafe NSW and WorkCover NSW; and medical retrieval and hospital care is provide by the NSW Ambulance Service, the Royal Flying Doctor Service of Australia, and the NSW Department of Health. If all of these agencies in only one state represent different facets of only one injury area, how then should injury prevention efforts be structured at a national level for the best and most cost effective results?

A 'whole of government' approach to the prevention of injuries is needed. Government agencies (and non-government agencies) must work collaboratively if the burden of injuries is to be significantly reduced [24]. A whole of government approach would ultimately reduce duplication, focus resources, promote the best use of skills and expertise, and encouraged sustainability of effort through the strengthening of cross-government networks and partnerships. However, the adoption of a whole of government approach to injury prevention would have implications for the design of government processes, as it would likely involve the integration of planning across government for injury prevention programs, the development of intergovernmental partnership agreements between agencies, the creation of a format for pooled resourcing for injury prevention efforts, the enhancement of injury surveillance practices, and the development of structured key cross-government performance indicators.

With the launch of the *National Injury Prevention and Safety Promotion Plan 2004–2014*[21] it is now of vital importance that this policy be progressed to include a whole of government commitment to establishing within each agency the process of cross-government collaboration needed to implement the plan.

In New Zealand, the implementation plan for the New Zealand Injury Prevention Strategy is the responsibility of the New Zealand Cabinet who report to the New Zealand Parliament [25]. In Australia, an implementation plan for the *National Injury Prevention and Safety Promotion Plan *[21] has not even been developed.

Ownership, commitment and national coordination of sustained injury prevention efforts needs to be at the highest level and it is recommended that the Council of Australian Governments (COAG) would be an ideal forum to monitor and discuss cross-government actions in the implementation of injury prevention policy. The COAG has previously discussed some aspects of injury prevention policy, including issues surrounding gun control, domestic violence, child protection, and marine safety[26]. It is recommended that the governance of the implementation of the National Injury Prevention and Safety Promotion Plan 2004–2014 [21] be placed with COAG. The formation of a cross-government Ministerial Council could assist in providing advise to COAG on injury prevention priority areas, best use of resources for cost-effective results and integration of planning of prevention activities. Supporting documentation on the incidence of injury in Australia, the development of performance measures and injury indicators could come from enhanced resourcing of already existing bodies, such as the National Injury Surveillance Unit.

Without the allocation of sufficient resources, and the development of an appropriate national governance and performance management structure as described above, the *National Injury Prevention and Safety Promotion Plan 2004–2014 *[21] is unlikely to meet its goals. Through the engagement of all sectors of government in a cross-government Ministerial Council and steerage by the peak intergovernmental forum, COAG, the impetus for national coordination of injury prevention efforts is strengthened and the commitment of all sectors of government to the reduction of injury is emphasised.

Ultimately, the measure of effectiveness of any national public health policy is a decrease in the population burden and associated costs of the relevant health condition. The dramatic decline in transport-related injuries since 1986 (18.0 per 100,000 population in 1986 to 7.9 per 100,000 population in 2004 [27]) demonstrates the scope for prevention in the injury field. This decline can be directly attributed to the National Road Safety Strategy, improvements in trauma management, and consequent substantial commitments at state and territory government levels by the departments of roads, transport and police. If the *National Injury Prevention and Safety Promotion Plan 2004–2014 *[21] is to lead to a decrease in the incidence of injury then similar effort needs to be mounted for non-transport related injury.

## Conclusion

Injuries are preventable. Yet, after two decades of national policy development, injuries remain the leading cause of death for Australians under the age of 44 [1]. The *National Injury Prevention and Safety Promotion Plan 2004–2014 *[21] has recently been endorsed by the Australian Health Ministers to guide a coordinated national response to the problem of injury. A concerted effort must now be made by the signatories to this national document to honour their commitment, as prescribed in the plan, to reducing the injury-related harm that is currently having a serious public health impact on individuals, the community, and the Australian economy.

## Declaration of competing interests

All information on which this article is based is in the public domain. It should be noted however that R McClure was co-chair of SIPP from 2002 to 2005 and R Mitchell was a member of SIPP during 2004.

The authors declare that they have no competing interests.

## Authors' contributions

RM prepared the first draft of the manuscript. RMc contributed to the development of ideas expressed in the manuscipt and assisted in preparing the final version. Both authors read and approved the final manuscipt.
